# Automatic Forecast of Intensive Care Unit Admissions: The Experience During the COVID-19 Pandemic in Italy

**DOI:** 10.1007/s10916-023-01982-9

**Published:** 2023-08-05

**Authors:** Danila Azzolina, Corrado Lanera, Rosanna Comoretto, Andrea Francavilla, Paolo Rosi, Veronica Casotto, Paolo Navalesi, Dario Gregori

**Affiliations:** 1 Department of Environmental and Preventive Sciences, University of Ferrara, Ferrara, Italy; 2 Unit of Biostatistics, Epidemiology, and Public Health, Department of Cardiac, Thoracic, Vascular Sciences and Public Health, University of Padova, Via Loredan, 18, Padova, 35131 Italy; 3 Department of Public Health and Pediatrics, University of Turin, Turin, Italy; 4 Institute of Anaesthesia and Intensive Care, Padua University Hospital, Padua, Italy; 5 Department of Medicine (DIMED), University of Padua, Padua, Italy

**Keywords:** ETS, COVID-19, ICU, Monitoring tool, Time Series, Forecast

## Abstract

The experience of the COVID-19 pandemic showed the importance of timely monitoring of admissions to the ICU admissions. The ability to promptly forecast the epidemic impact on the occupancy of beds in the ICU is a key issue for adequate management of the health care system.

Despite this, most of the literature on predictive COVID-19 models in Italy has focused on predicting the number of infections, leaving trends in ordinary hospitalizations and ICU occupancies in the background.

This work aims to present an ETS approach (Exponential Smoothing Time Series) time series forecasting tool for admissions to the ICU admissions based on ETS models. The results of the forecasting model are presented for the regions most affected by the epidemic, such as Veneto, Lombardy, Emilia-Romagna, and Piedmont.

The mean absolute percentage errors (MAPE) between observed and predicted admissions to the ICU admissions remain lower than 11% for all considered geographical areas.

In this epidemiological context, the proposed ETS forecasting model could be suitable to monitor, in a timely manner, the impact of COVID-19 disease on the health care system, not only during the early stages of the pandemic but also during the vaccination campaign, to quickly adapt possible preventive interventions.

**Supplementary Information:**

The online version contains supplementary material available at 10.1007/s10916-023-01982-9.

## Introduction

The COVID-19 pandemic first appeared in China in late 2019 [1]. Since then, the epidemic has spread throughout the world, causing a considerable number of deaths [2]. Furthermore, the virus has caused an overload of hospital systems, especially intensive care units (ICUs) in many countries [3].

Italy, in particular, is the first European country in which the outbreak of the COVID-19 epidemic has spread since the end of February 2020 [4]. In the so-called first phase of the epidemic, the infection spread mainly in northern regions such as Lombardy, Veneto, Piedmont, and Emilia-Romagna [5].

Measures to contain the COVID-19 outbreak, including social distancing, business and school closures, and temporary travel bans, have been implemented since February 2020. These policies were initially introduced in the northern regions and later extended to the whole country [6]. The national lockdown contained the spread of the virus throughout the territory almost completely, halting the progression of the infection throughout the summer period of 2020 [7].

The COVID-19 epidemic resumed its run, involving all Italian regions, from October 2020 [7]. On March 2021, there were 101,881 cumulative COVID deaths in Italy, with 2982 beds occupied in the ICU [8].

From then on, Italy has adopted diversified containment policies according to regions, due to the autonomy of the single region in deciding on health issues. According to the latest government measures, Italy is divided into three risk zones: high-risk (red zone), intermediate (orange zone), and low-risk (yellow zone) [9]. Different rules and prohibitions correspond to different colors. The classification of a region in one of the three risk zones is decided by the Ministry of Health according to 21 criteria. One of the most relevant criteria is the occupancy situation of the hospital and the ICU bed [9], together with the current number of Rt reproduction numbers [10] and the presence of local outbreaks of infection.

The experience of the COVID-19 pandemic in Italy showed the importance of timely monitoring of admissions to the ICU [11]. The shortage of beds in the ICU can result in a trade-off between saving one patient’s life over another; therefore, the ability to quickly forecast the epidemic impact on the occupancy of beds in the ICU is a key issue for adequate management of the healthcare system during an emergency [12]. Timely forecasting of ICU occupancy levels is essential to adjust ICU capacity to meet demand or plan patient transfer efforts [13].

Despite this, most of the literature on predictive COVID-19 outbreak models in Italy has focused on predicting the number of infections, leaving trends in ordinary hospitalizations and ICU occupancies in the background.

Stochastic-compartmental models (SI, SIR, SEIRD) [14, 15] are widely applied to predict the spread pattern of the disease focusing on public health interventions to limit the spread of the pandemic [16]. However, these models are based on assumptions derived from validated information on virus transmission mechanisms [16]. These stochastic-compartmental methods are widely applied to model the spreading diffusion of the epidemic but are little used to monitor ICU occupancy.

Classical prediction approaches such as exponential, Poisson, and logistic models, have been widely applied to characterize the spread of disease during the early stages of the epidemic [17]. Classical models are also considered in the literature to predict admissions to ICU admissions in Italy in the early stages of the pandemic [13, 18]. Exponential models show unreasonable predictions in the late stages of the pandemic. Alternatively, logistic-related growth models revealed a more suitable fit for the late stages of the epidemic, as disease spread begins to decelerate as it approaches the maximum capacity limit [19].

Other prediction models are based on the ARIMA (Auto-Regressive Integrated Moving Average) time-series forecasting technique. These methods, unlike classical models, have improved performance in adjusting estimates to time-series fluctuations in different stages of the epidemic [20–23]. These models deal with both seasonal and non-seasonal time series.

The model selection procedure can be performed in an automated way in an ARIMA model to maximize the forecast accuracy [24]. These time series models have been applied in the literature to develop predictive ICU occupancy tools in Italy [25].

Other time-series parameterizations are Exponential Smoothing Time-Series models (ETS). One of the advantages of this technique is that it provides a higher weighting of estimates in the correspondence of observations closer to the forecast time [26]. ETS models make seasonal adjustments easier, handle multiple time series of seasonality, and are flexible enough to handle noninteger seasonal periods [27].

An important issue to deal with in a time series analysis is the stationarity assumption. The probabilistic structure of stationary time series satisfies certain conditions of time invariance. For example, parameters such as mean and variance do not change over time [28]. The literature demonstrated that in several research fields, this assumption could not be proven. In this regard, ETS models, unlike some ARIMA parameterizations, appropriately handle nonstationary time series [29].

However, ETS models have little been applied in the literature to forecasting COVID-19 ICU occupancy trends, and only one research article shows that these models also outperform classical time series models for predictions of the trends of the COVID-19 epidemic in Italy [30].

This work aims to present a time series forecasting tool for admissions to the ICU based on ETS models. The results of the forecasting model are presented for the regions most affected by the epidemic, such as Veneto, Lombardy, Emilia-Romagna, and Piedmont [31].

## Materials and Methods

The prediction of the ETS model was developed according to regions using the official data on admissions to the ICU admissions published by the Civil Protection Department [8].

The prediction models are available, for all Italian regions, on the COVID-19ita [32] website (https://r-ubesp.dctv.unipd.it/shiny/covid19ita/) created by the COVID-19-Ita research group (Unit of Biostatistics, University of Padua, Italy). The proposed tool is available in the ICU Regional Monitoring Section [33].

The same predictions have been published on the official platform of the Italian agency AGENAS (National Agency for Regional Health Services) (https://www.agenas.gov.it/covid19/web/index.php?r=site/index).

### ETS Model

The ETS (Error, Trend, Seasonality) models are widely used for time series forecasting during the pandemic period [34]. This model leads to identifying and quantifying different components that contribute to the fluctuation in the observed ICU occupancy data. These components are error, trend, and seasonality. This parametrization provides a framework for making predictions and forecasts regarding ICU admissions [35]. The models utilize a weighted average of past ICU admissions to predict future values by assigning more relevance to recent observations in the time series, resulting in a decreasing exponential weighting of older observations [36].

1. Error: the error component accounts for random fluctuations or unexpected variations in the pandemic data. It represents the deviation between the observed values and the predicted values [35].
2. Trend: the trend component captures the long-term direction or pattern observed in the ICU entrance data. This component leads to identifying whether the data is increasing, decreasing, or exhibiting a more complex pattern over time. By understanding the pandemic trend, epidemiologists can gain insights into the overall progress or changes in the disease evolution and ICU occupancy [34].
3. Seasonality: the seasonality component deals with recurring patterns or cycles that occur within specific time intervals. In epidemiological settings, these cycles could be related to seasonal effects, such as fluctuations in diffusion or disease prevalence that occur at certain times of the year. The seasonality can aid clinicians, epidemiologists, and decision-makers in understanding the timing and potential triggers of the pandemic diffusion [35].

To apply the ETS model it is useful to select the appropriate configuration based on the characteristics of the patient data. This involves determining whether the error, trend, and seasonality components should be added, multiplied, or excluded from the model. The error component can be additive (A) or multiplicative (M). The trend component can be additive (A), additive damped (Ad) specifically for the trend, or could be excluded (N). Similarly, the seasonality component can be additive (A), multiplicative (M), or excluded (N) from the model [37].

The possible combinations for each model component are as follows:

1. Error ={A, M};
2. Trend = {N, A, Ad};
3. Seasonal = {N, A, M}.

For example, let’s consider the ETS(A, Ad, N) model, which represents an exponential time series smoothing approach with additive error, additively damped trend, and no seasonality components. The damped trend method assumes the presence of a trend in the time series but expects that the pandemic growth rate observed at the end of the historical data will not continue for an extended period into the future [38]. The introduction of a damping parameter slows down the trend, leading to a nonlinear trend component [37].

The model is mathematically composed of three equations: (1) a level equation, and (2) a growth equation with a common source of additive error ${\epsilon }_{t}$[36]. These two components are combined in the (3) forecast equation to generate predictions for future periods.

• Level equation ${g}_{t}$ at *t* time:${g_t}\, = \,{g_{t - 1}}\, + \,\phi {b_{t - 1}}\, + \,\alpha {\varepsilon _t}$

• Growth equation ${b}_{t}$ at *t* time:${b}_{t}=\varphi {b}_{t-1}+\beta {\epsilon }_{t}$

• Forecast equation:$${y}_{t}={g}_{t-1}+\varphi {b}_{t-1}+{\epsilon }_{t}$$

Therefore, the growth for the one-step forecast of ${y}_{t}$ is $\varphi {b}_{t-1}$, and the growth is dampened by a factor of $\varphi$ for each additional future period. Instead, the values $\alpha$and $\beta$, are smoothing constants [39].

The supplementary material provides detailed taxonomies [38] for additive error models (Table S1) and multiplicative error models (Table S2).

### Model Selection Procedure

The parameters of the ETS model are estimated using a maximum likelihood approach [36]. The likelihood is the probability of the data that arises from the specified model. A higher likelihood is associated with a good model [40].

Likelihood-based approaches are also considered for the ETS model selection criteria [36]. The widely used approaches are the Akaike Information Criterion (AIC), the small sample corrected Akaike Information Criterion (AICc), and the Bayesian Information Criterion (BIC) [41]. The BIC criterion, which accounts for the time series length, was considered for the automatic model selection procedure. Automatically identified changes in model parameterization have been reported for the selected regions considering ETS models parameterized on a daily growing fraction of the time series.

### Predictive Model Assessment

A predictive model assessment has been considered using a proper scoring rule procedure, adapted to count data [42]. This rule leads to the simultaneous evaluation of the model calibration and sharpness summarized in a single score [43]. The calibration property refers to the statistical consistency between the predictive distribution and the time-series data, while the sharpness refers to the concentration of the predictive distribution and is a property of the forecasted values. The more concentrated the predictive distribution, the sharper the forecasts obtained [43].

### Proper Scoring Rule

A proper score$s\left({P}_{t},{Y}_{t}\right)$ is a function of the observed data ${Y}_{t}$and a predictive cumulative density function (CDF) ${P}_{t}$. The author assumed a Poisson or negative binomial CDF for the general estimation of the proper scoring rule of the count data [44].

The Mean Squared Error Score (SES), defined as$\text{s}\text{e}\text{s}\left({P}_{t},{Y}_{t}\right)={\left({Y}_{t}-{\mu }_{{P}_{t}}\right)}^{2}$, has been considered for the predictive model because it does not depend on distribution assumptions. SES is identical for both the Poisson and negative binomial distribution assumption since the conditional means ${\mu }_{{P}_{t}}$ are equal for both distributions [45].

The mean SES has been estimated on a sequentially daily growing fraction of time series data from 10 to 2020 until 18 December 2021. A local polynomial regression smoothing (LOESS) has been estimated in the point data with a span of 0.75 and a degree of approximation equal to 2.

The Root Mean Square Error (RMSE), defined as$RMSE=\sqrt{{\left({Y}_{t}-{\widehat{Y}}_{t}\right)}^{2}}$ ( ${\widehat{Y}}_{t}$are the estimated time series value) has been calculated together with the Mean Square Error (MSE) ($MSE={\left({Y}_{t}-{\widehat{Y}}_{t}\right)}^{2}$) on the sequentially growing fraction of time daily.

### Time-series Assessment via Cross-Validation Procedure

The regional time series have been divided into 94 times of weekly data. The model has been trained in each fold and validated in the remaining time series. The median and interquartile range (IQR) performance metrics across folds have been computed using SES, RMSE MSE, and Mean Absolute Percent Error (MAPE), defined as$MAPE=\sum _{t=1}^{n} \left|\frac{{Y}_{t}-{\widehat{Y}}_{t}}{{Y}_{t}}\right|$

### Time-series Regularization

The Box-Cox transformation procedure, one of the most applied methods used to regulate count data, often characterized by the presence of heteroskedasticity regarding time, has been applied for time series regularization [46, 47]. Guerrero’s method [48] has been used to define an optimal regularization parameter () that minimizes the coefficient of variation within the data.

## Results

### Epidemic Pattern Description

**Fig. 1:**
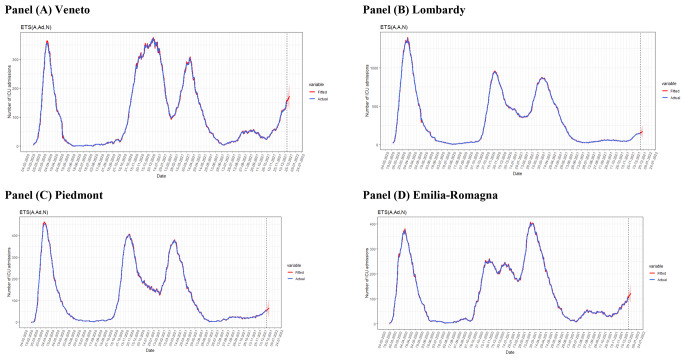
ETS predictions for admissions to the ICU. The trend of observed (blue line) and expected (red line) ICU admissions has been reported. The 7-day predictions from the ETS model have also been also shown together with the 95% confidence intervals (small light-red area). On the plots, also the ETS parametrizations were selected via BIC criterion

Figure 1 reports the number of admissions to the ICU observed (blue lines) and estimated by ETS (red lines) from March 1st 2020 to December 18th 2021. The overall trend pattern is similar for all regions analyzed.

The absolute numbers of admissions to the ICU in Lombardy were higher throughout the epidemic compared to other regions. During the first wave, all regions had a peak in admissions in ICU around the end of March, and then a rapid decrease to almost zero admissions was observed in the summer of 2020 (Fig. 1).

In contrast, the trends that characterize the epidemic since October 2020 (second wave) have a different pattern between regions.

In the Veneto region (Fig. 1, panel A), for example, the phenomenon reached a peak on January 1st, 2021 with 372 admissions to the ICU, similar to the first peak of 356 admissions registered on March 30th, 2020. The number of admissions remained high and close to the maximum value from mid-November to mid-January 2021. The effect of the epidemic on ICU occupancy decreased until the middle of February, with a net increase in March 2021.

In the Lombardy region (Fig. 1, Panel B), on the other hand, the second wave of the epidemic presented a peak of 942 admissions to the ICU on November 22nd, 2021. In this setting, a continuous decrease was also observed until February 2021. In March, another resurgence of the effects of the infection on ICU admissions was registered.

The pattern of admissions to the ICU in the second wave in the Piedmont region (Fig. 1, Panel C) was similar to that in Lombardy. The region experienced the first peak of ICU admissions on November 24th 202 (404 cases) and then a decrease in the phenomenon until a new peak in March 2021.

Finally, in the Emilia-Romagna region (Fig. 1, Panel D), the number of admissions to the ICU increased in the second wave to approximately 200–250 cases for a long period ranging from November 2020 to mid-February 2021. In March 2021, the spread of the epidemic led to a rapid increase in ICU admissions up to 402 cases on March 25th, 2021, exceeding the peak observed during the first wave on April 5th, 2020 (375 admissions).

A subsequent general decline in both the overall epidemic pattern and its impact on admissions to the ICU was registered in all regions considered from April 2021 until the end of October. At that time, the number of admissions to the ICU admissions increased throughout November-December 2021, especially in the Veneto and Emilia-Romagna regions (Fig. 1, Panel A and D), although at notably lower levels compared to the same period in 2020.

### Forecasting

The 7-day forecasts indicate an increase in admissions to the ICU for all selected regions (Fig. 1, red lines). The increase is most marked in the Veneto and Emilia-Romagna regions. The situation for Piedmont seems to be oriented toward the stabilization of the phenomenon.

### ETS Parameterizations

Concerning the ETS estimation, the BIC selected parameterizations are ETS(A, Ad, N) for all the regions except for Lombardy, for which the optimal model is an ETS(A, A, N). The additive error and the absence of seasonality is a common issue in all the ETS parameterizations (Fig. 1).

**Fig. 2:**
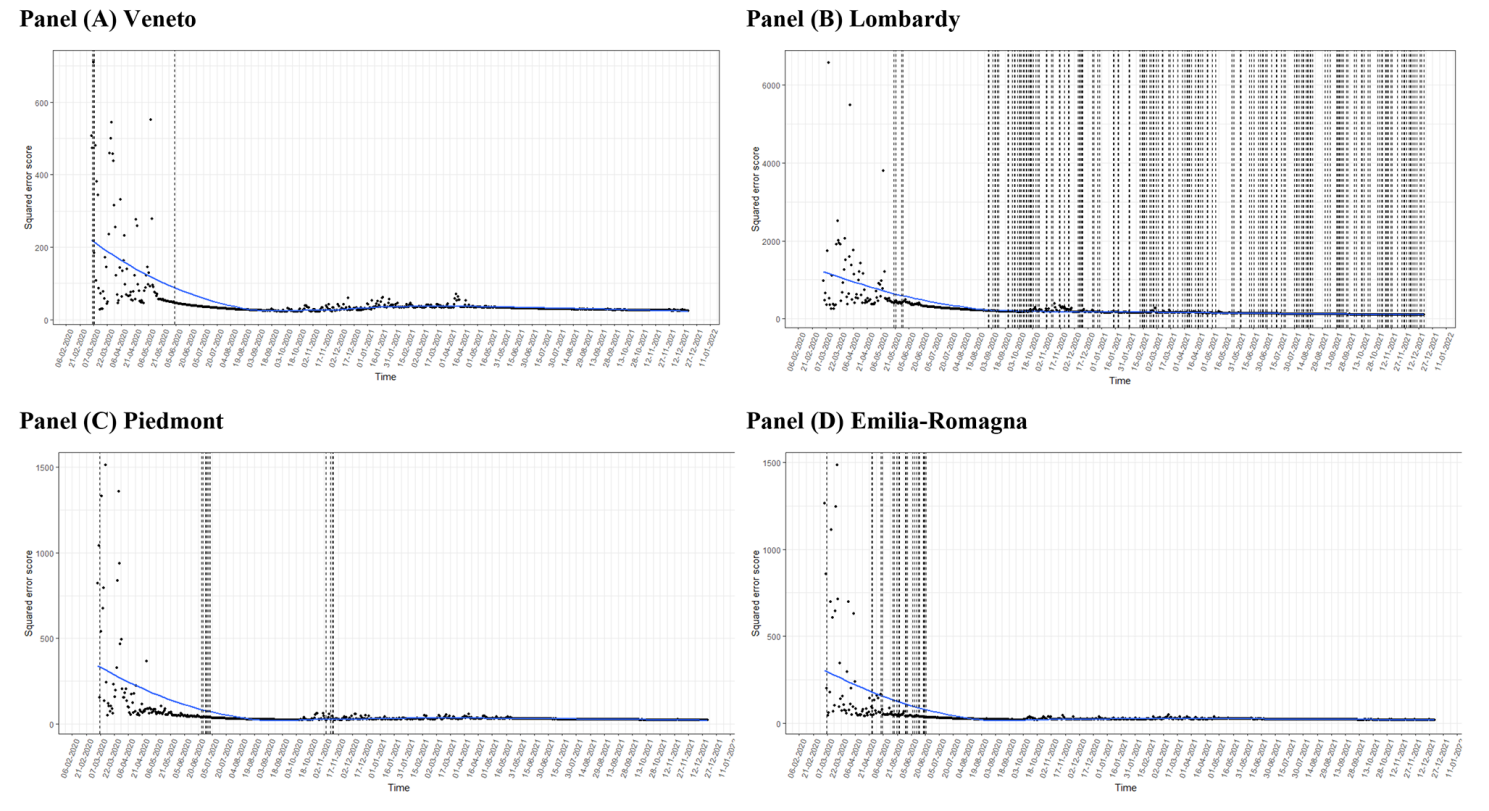
The squared error score (SES) has been estimated on a sequentially daily growing fraction of time series data from 10 of March 2020 until the 10 March 2021. A local polynomial regression smoothing (LOESS) has been estimated in the point data with a span of 0.75 and a degree of approximation equal to 2. The vertical dotted lines represent the changes in the model parameterizations

For all regions, after an initial adaptation phase, a change in parameterization is observed around May-June 2020; the trend in that period smoothes out from additive to additive damped (Table S3). The Lombardy region records the highest number of parameter changes, essentially alternating between an additive and a damped additive trend (Table S3, Fig. 2, Figure S1, Figure S2).

### ETS Performance

For all selected regions, the SES decreases considering increasing fractions of the time series (Fig. 2) and tends to stabilize from December 2020. A minimal increase in error is also observed during the first signs of the resurgence of the epidemic at the end of October 2020, especially in the Emilia-Romagna region (Fig. 2). A similar pattern is also observed when considering RMSE (Figure S1) and MSE (Figure S2).

According to the information criteria calculated on the overall time series, the BIC is generally higher than the AIC and AICc and higher for the Lombardy and Veneto regions (Table 1). Regarding cross-validated error measures, SES, RMSE, and MSE are similar in the Veneto, Emilia-Romagna, and Piedmont regions, with a three-fold pattern lower than those calculated for the Lombardy region. In contrast, the percentage error (MAPE) is lower for the Lombardy region compared to the others (Table 2).

**Table 1:** Information criteria of the final ETS estimated model according to metrics and regions

	Veneto	Emilia-Romagna	Piedmont	Lombardy
BIC	6488	6339	6404	7424
AIC	6461	6312	6377	7401
AICc	6462	6312	6377	7402

Abbreviations: BIC (Bayesian Information Criterion); AIC (Akaike Information Criterion); AICc (Akaike Information Criterion, corrected).

**Table 2:** ETS (94 weekly folds) according to regions. The median and interquartile range (IQR) has been reported

	Veneto	Emilia-Romagna	Piedmont	Lombardy
SES	40 (153)	38 (74)	36 (141)	114 (647)
RMSE	6 (10)	6 (7)	5 (10)	12 (25)
MSE	38 (171)	39 (105)	24 (150)	149 (871)
MAPE	11 (17)	8 (12)	9.5 (11)	7 (11)

Abbreviations SES (Squared Error Score), RMSE (Root Mean Square Error), MSE (Mean Square Error); MAPE (Mean Absolute Percentage Error

## Discussion

This research presents a time series forecasting tool for COVID-19 ICU admissions based on ETS models. Estimation of time series of ETS has been reported for the regions most affected by pandemics since the virus first appeared in Italy.

COVID-19 exploded in Northern Italy with the greatest impact in Lombardy, where the first outbreak took hold [49]. This analysis confirmed that the absolute observed and estimated absolute values of admissions to the COVID-19 ICU admissions, during the pandemic period, are constantly higher for Lombardy compared to the other considered regions (Veneto, Emilia-Romagna, Piedmont).

Regarding the time series pattern, all regions had a peak in ICU admissions around the end of March and then observed a rapid decrease to almost zero admissions in the summer of 2020. This common trend probably reflects national health measures, where the decision-making power of a single region was marginal during the first wave. Containment policies were initially introduced in February 2020 in northern regions and were later extended to the whole country [6]. The national lockdown contained the spread of the virus throughout the territory almost completely halting the progression of the infection throughout the summer period of 2020 [7].

In October 2020, as the pandemic spread again, prevention policies were differentiated by region and defined according to indicators of epidemic progression. All this is reflected in a heterogeneous second-phase trend of ICU occupancy in the Italian regions.

Lombardy and Piedmont, for example, were immediately subjected, as of October 26, 2020 [50], to more restrictive measures being placed in the so-called red zone, where movements are limited only to work and emergency reasons. This led to a peak in admissions to the ICU admissions that lasted until the end of November, followed by a sudden reduction in admissions throughout December.

A long-term policy effect is evidenced in the reduction of the ICU from 26 to 2020. This is supposed to be explained by the fact that the median time between symptoms onset and admission to symptoms onset and ICU is approximately 11 (IQR 8-14) [51] days, to which a median incubation time of 5.1 days (95% CI: 4.5–5.8) days [52].

Lombardy and Veneto are the Italian centers of excellence in healthcare facilities [53]. The Lombardy region has a higher number of intensive care and resuscitation beds; unfortunately, these hospitals are rapidly running out of hospital beds for the provision of primary care for conditions other than COVID-19 [53].

The Veneto region, on the other hand, has adopted policies less restrictive compared to Lombardy during the second wave of the pandemic, probably due to the prompt response of the regional health system [54]. The region remained in the yellow zone from October 26th until mid-March [50]. The stores and activities remained open during the day and restrictions were imposed on movement during the evening hours [55]. This probably led to a peak in ICU admissions at levels comparable to the first wave, which lasted until the second half of January. Since then, the epidemic has begun to slow its effects on ICU entrances.

In particular, the second wave in Emilia-Romagna carried a less empathic peak, a lower resolution, and almost no transition between the second and subsequent third surge in infections and the number of admissions to the ICU. Like the Veneto region, Emilia-Romagna has also been in the yellow zone for a long time, with a consequent long-wave effect on the number of ICU accesses [56].

In January 2021, many of the restrictions were relaxed, and almost all regions moved into the yellow zone [56]. After this period, from the end of February 2021, there was an increase in the number of admissions to the COVID-19 ICU admissions in all the regions considered, with estimates and forecasts tending to an increasing pattern.

Following the recovery of the epidemic in the summer of 2021, a resurgence of the epidemic and its impact on the ICUs is observed starting in October. The impact is more limited than in the previous year but is still increasing, especially in Veneto and Emilia-Romagna. The effect is more limited because the coverage of COVID vaccination with at least two doses in Italy is currently at December 18th at 79.9%, with few differences between the regions considered (Veneto 79.7%, Piedmont 78.6, Lombardy 81.6%, Emilia-Romagna 82.5%) [57–59].

The aforementioned history of the epidemic’s evolution, with its variations and rapid increases and decreases, makes it difficult for the healthcare system to adapt [60]. The lesson learned in these situations is that monitoring bed occupancy in the ICU is crucial to avoid situations of health system overload [14]. This is evidenced especially during the expansive phases of the epidemic [60]. For this reason, simple, data-driven methods are useful that may provide accurate and timely forecasts of hospital bed demand [13].

The literature has shown that ETS-type time series models perform well in predicting ICU occupancy in the short term. Our results, along the same lines, show optimal performance for the predictive tool [30]. The predictive error decreases as the length of the series increases and stabilizes after six months for all regions considered. Moreover, other applications of the ETS-type smoothing model have been shown to predict the optimal number of ICU beds to reduce patient waiting time even in ordinary pre-COVID-19 management situations [61].

As a result, our forecasting tool, implemented on the COVID-19ita [32] website, provides stable estimates, not only in the more advanced stages of the epidemic but also during the vaccination campaign. Taking into account this general framework, an ICU occupancy forecasting tool, customized to regional healthcare systems, is useful to monitor the pandemic situation facilitating the timely adoption of appropriate measures and prevention policies to avoid the uncontrolled impacts of the COVID-19 epidemic on hospital facilities.

## Conclusions

The structural load monitoring process in the ICU has proven to be of great importance in the different phases of the COVID-19 pandemic. The lesson learned is that flexible and up-to-date forecasting tools could be useful to follow the evolution of the pandemic at the macro territorial level, especially when containment policies are defined at regional levels and are quickly adapted to follow the evolution of epidemic diffusion.

### Electronic Supplementary Material

Below is the link to the electronic supplementary material.

Supplementary Material 1
