# Supplementary material for: Automatic Forecast of Intensive Care Unit Admissions: The Experience During the COVID-19 Pandemic in Italy
Source: J Med Syst. 2023 Aug 5;47(1):84. doi: 10.1007/s10916-023-01982-9 (PMC10404188; doi:10.1007/s10916-023-01982-9)
Supplement: Supplementary file 1 — Supplementary Material 1 [file 10916_2023_1982_MOESM1_ESM.docx]

**Supplementary material**

**Table S1. ETS taxonomy for additive error models.** The time series level at the time t$is g_{t}$; the seasonal component at time t is $s_{t};$the order of seasonality is defined by m. The smoothing parameters are

|  | **Seasonal** | | |
| --- | --- | --- | --- |
| **Trend** | **N** | **A** | **M** |
| **N** | $\begin{matrix} y_{t}=\text{g}_{t-1}+\varepsilon_{t} \\ g_{t}=g_{t-1}+\alpha\varepsilon_{t} \end{matrix}$ | $\begin{matrix} y_{t}=g_{t-1}+s_{t-m}+\varepsilon_{t} \\ g_{t}=g_{t-1}+\alpha\varepsilon_{t} \\ s_{t}=s_{t-m}+\gamma\varepsilon_{t} \end{matrix}$ | $\begin{matrix} y_{t}=g_{t-1}s_{t-m}+\varepsilon_{t} \\ g_{t}=g_{t-1}+\alpha\varepsilon_{t}/s_{t-m} \\ s_{t}=s_{t-m}+\gamma\varepsilon_{t}/g_{t-1} \end{matrix}$ |
| **A** | $\begin{matrix} y_{t}=g_{t-1}+b_{t-1}+\varepsilon_{t} \\ g_{t}=g_{t-1}+b_{t-1}+\alpha\varepsilon_{t} \\ b_{t}=b_{t-1}+\beta\varepsilon_{t} \end{matrix}$ | $\begin{matrix} y_{t}=g_{t-1}+b_{t-1}+s_{t-m}+\varepsilon_{t} \\ g_{t}=g_{t-1}+b_{t-1}+\alpha\varepsilon_{t} \\ b_{t}=b_{t-1}+\beta\varepsilon_{t} \\ s_{t}=s_{t-m}+\gamma\varepsilon_{t} \end{matrix}$ | $\begin{matrix} y_{t}=\left(g_{t-1}+b_{t-1}\right)s_{t-m}+\varepsilon_{t} \\ g_{t}=g_{t-1}+b_{t-1}+\alpha\varepsilon_{t}/s_{t-m} \\ b_{t}=b_{t-1}+\beta\varepsilon_{t}/s_{t-m} \\ s_{t}=s_{t-m}+\gamma\varepsilon_{t}/\left(g_{t-1}+b_{t-1}\right) \end{matrix}$ |
| **A_d_** |  |  |  |

**Table S2. ETS taxonomy for multiple error models.** The time series level at the time t$is g_{t}$; the seasonal component at time t is $s_{t};$the order of seasonality is defined by m. The smoothing parameters are

|  | **Seasonal** | | |
| --- | --- | --- | --- |
| **Trend** | **N** | **A** | **M** |
| **N** | $\begin{matrix} y_{t}=g_{t-1}\left(1+\varepsilon_{t}\right) \\ g_{t}=g_{t-1}\left(1+\alpha\varepsilon_{t}\right) \end{matrix}$ | $\begin{matrix} y_{t}=\left(g_{t-1}+s_{t-m}\right)\left(1+\varepsilon_{t}\right) \\ g_{t}=g_{t-1}+\alpha\left(g_{t-1}+s_{t-m}\right)\varepsilon_{t} \\ s_{t}=s_{t-m}+\gamma\left(g_{t-1}+s_{t-m}\right)\varepsilon_{t} \end{matrix}$ |  |
| **A** | $\begin{matrix} y_{t}=\left(g_{t-1}+b_{t-1}\right)\left(1+\varepsilon_{t}\right) \\ g_{t}=\left(g_{t-1}+b_{t-1}\right)\left(1+\alpha\varepsilon_{t}\right) \\ b_{t}=b_{t-1}+\beta\left(g_{t-1}+b_{t-1}\right)\varepsilon_{t} \end{matrix}$ | $\begin{matrix} y_{t}=\left(g_{t-1}+b_{t-1}+s_{t-m}\right)\left(1+\varepsilon_{t}\right) \\ g_{t}=g_{t-1}+b_{t-1}+\alpha\left(g_{t-1}+b_{t-1}+s_{t-m}\right)\varepsilon_{t} \\ b_{t}=b_{t-1}+\beta\left(g_{t-1}+b_{t-1}+s_{t-m}\right)\varepsilon_{t} \\ s_{t}=s_{t-m}+\gamma\left(g_{t-1}+b_{t-1}+s_{t-m}\right)\varepsilon_{t} \end{matrix}$ | $\begin{matrix} y_{t}=\left(g_{t-1}+b_{t-1}\right)s_{t-m}\left(1+\varepsilon_{t}\right) \\ g_{t}=\left(g_{t-1}+b_{t-1}\right)\left(1+\alpha\varepsilon_{t}\right) \\ b_{t}=b_{t-1}+\beta\left(g_{t-1}+b_{t-1}\right)\varepsilon_{t} \\ s_{t}=s_{t-m}\left(1+\gamma\varepsilon_{t}\right) \end{matrix}$ |
| **A_d_** |  | . |  |

**Figure S1.** The root mean square error (RMSEA) has been estimated on a sequentially daily growing fraction of time series data from 10 of March 2020 until the 18 December 2021. A local polynomial regression smoothing (LOESS) has been estimated in the point data with a span of 0.75 and a degree of approximation equal to 2. The vertical dotted lines represent the changes in the model parameterizations.

| **Veneto**  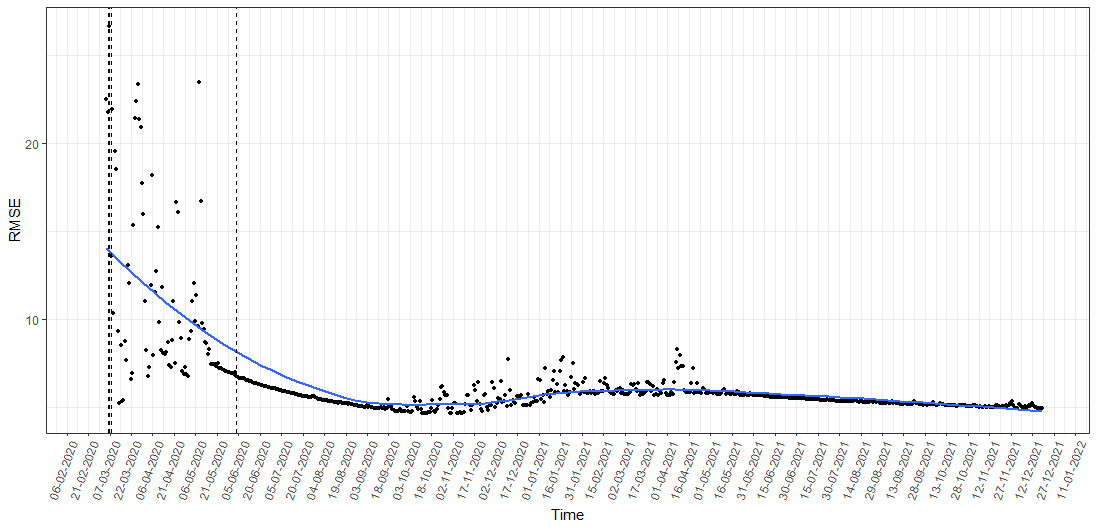 | **Lombardia**  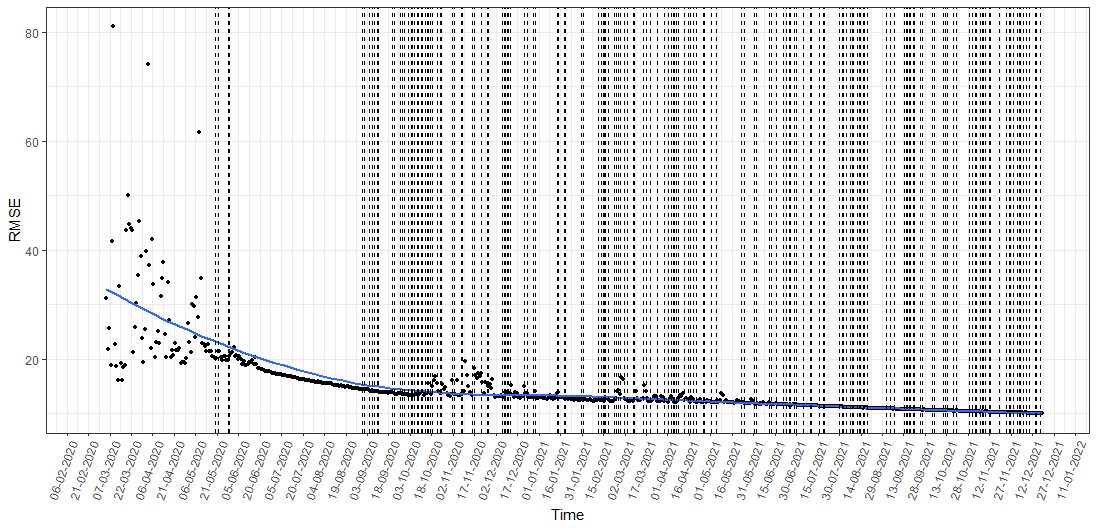 |
| --- | --- |
| **Piemonte**  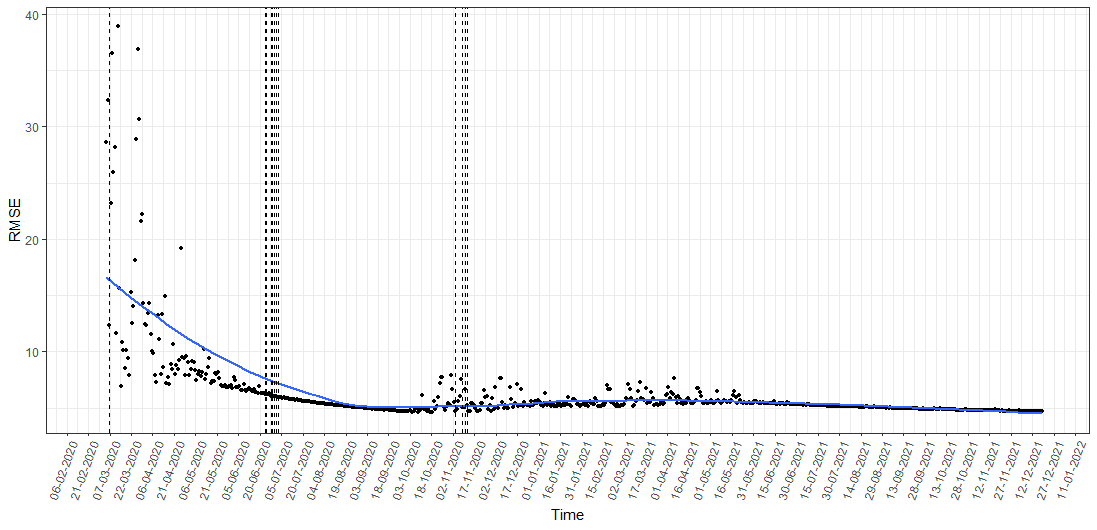 | **Emilia-Romagna**  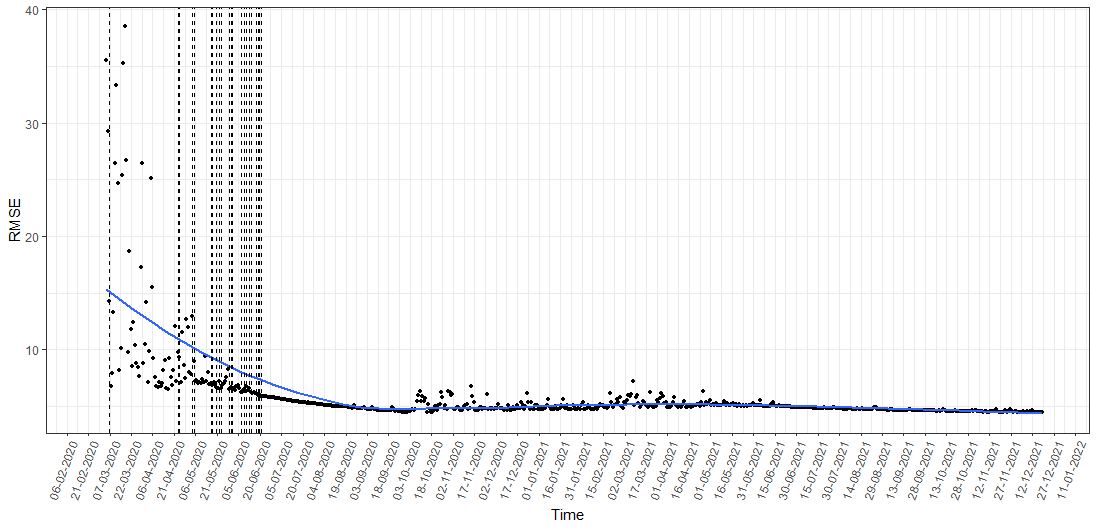 |

**Table S2.** The Mean Square Error (MSE) has been estimated on a sequentially daily growing fraction of time-series data from the 10 of March 2020 until the 18 of December 2021. A local polynomial regression smoothing (LOESS) has been estimated in the point data with a span of 0.75 and a degree of approximation equal to 2. The vertical dotted lines represent the changes in the model parametrizations.

| **Veneto**  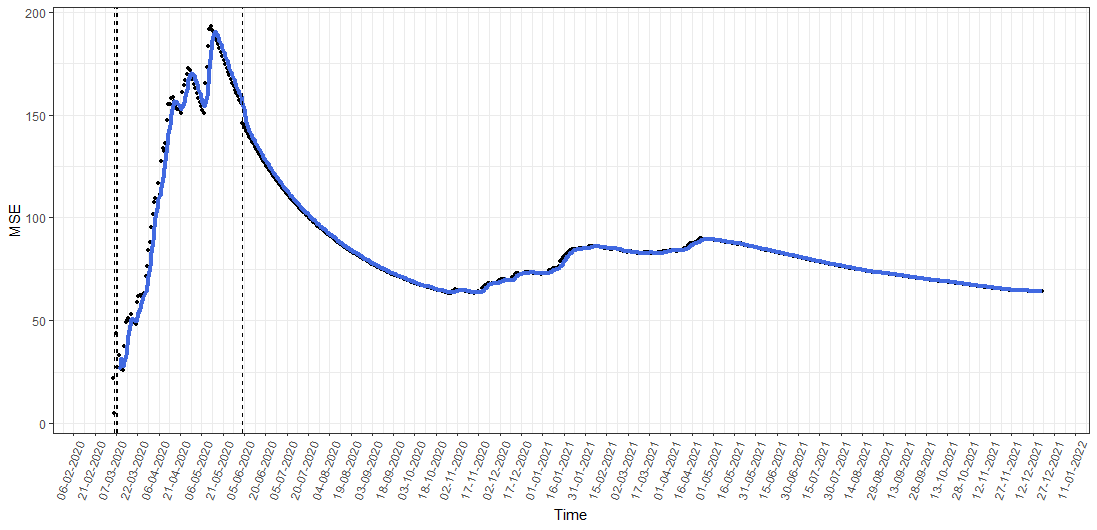 | **Lombardia**  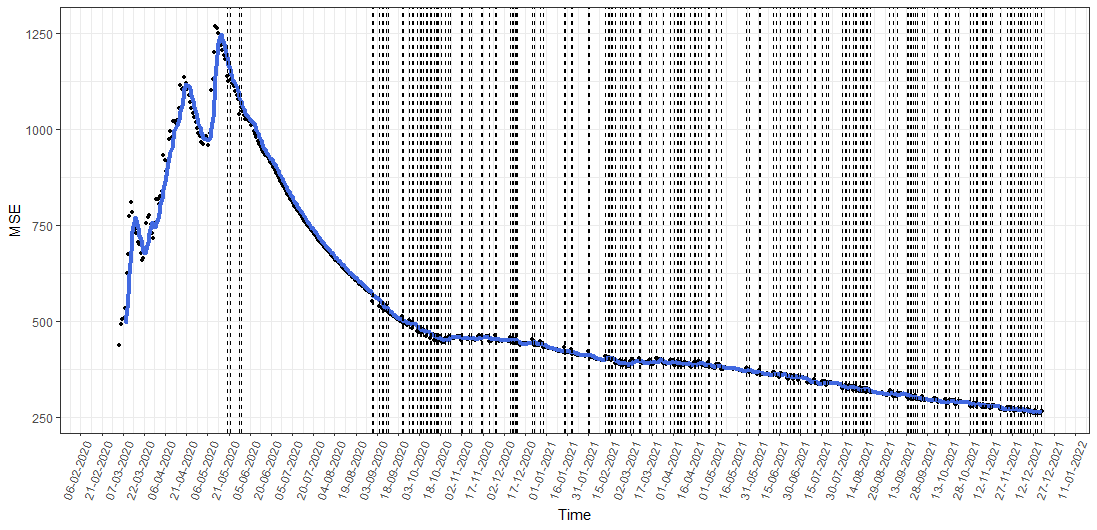 |
| --- | --- |
| **Piemonte**  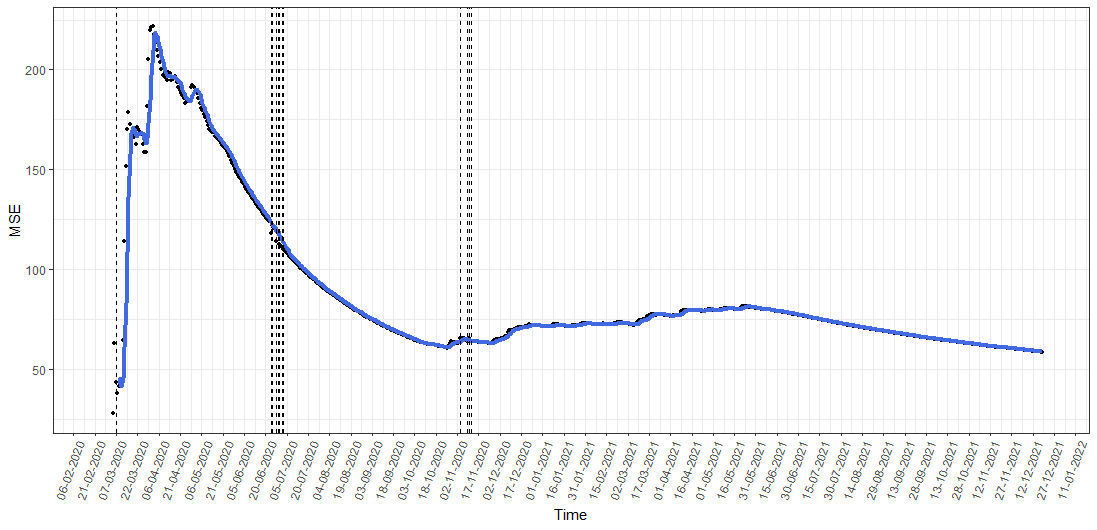 | **Emilia-Romagna**  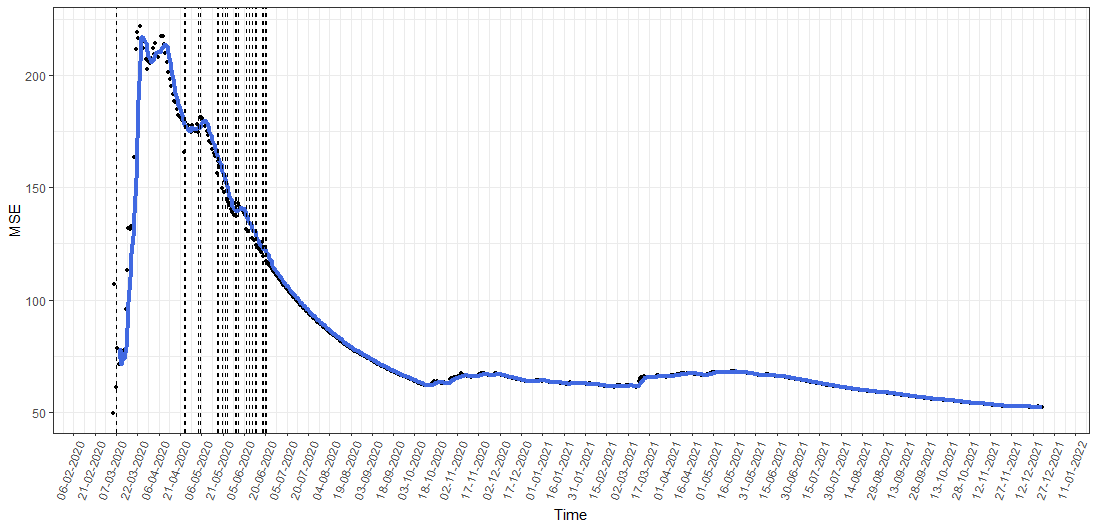 |

**Table S3.** Changes in the parametrization of the ETS model according to regions and identified days

| Veneto | | Emilia | | Piemonte | | Lombardia | |
| --- | --- | --- | --- | --- | --- | --- | --- |
| Dates | **Changes**  **in parametrization** | **Dates** | **Changes**  **in parametrization** | **Dates** | **Changes**  **in parametrization** | **Dates** | **Changes**  **in parametrization** |
| 05/03/2020 | ETS(A,A,N) | 06/03/2020 | ETS(A,A,N) | 06/03/2020 | ETS(A,A,N) | 19/05/2020 | ETS(A,Ad,N) |
| 06/03/2020 | ETS(A,N,N) | 23/04/2020 | ETS(A,Ad,N) | 23/06/2020 | ETS(A,Ad,N) | 21/05/2020 | ETS(A,A,N) |
| 07/03/2020 | ETS(A,A,N) | 24/04/2020 | ETS(A,A,N) | 24/06/2020 | ETS(A,A,N) | 28/05/2020 | ETS(A,Ad,N) |
| 03/06/2020 | ETS(A,Ad,N) | 03/05/2020 | ETS(A,Ad,N) | 27/06/2020 | ETS(A,Ad,N) | 29/05/2020 | ETS(A,A,N) |
|  |  | 04/05/2020 | ETS(A,A,N) | 28/06/2020 | ETS(A,A,N) | 30/08/2020 | ETS(A,Ad,N) |
|  |  | 16/05/2020 | ETS(A,Ad,N) | 29/06/2020 | ETS(A,Ad,N) | 31/08/2020 | ETS(A,A,N)** |
|  |  | 17/05/2020 | ETS(A,A,N) | 01/07/2020 | ETS(A,A,N) |  |  |
|  |  | 20/05/2020 | ETS(A,Ad,N) | 02/07/2020 | ETS(A,Ad,N) |  |  |
|  |  | 22/05/2020 | ETS(A,A,N) | 03/11/2020 | ETS(A,A,N) |  |  |
|  |  | 23/05/2020 | ETS(A,Ad,N) | 08/11/2020 | ETS(A,Ad,N) |  |  |
|  |  | 29/05/2020 | ETS(A,A,N) | 10/11/2020 | ETS(A,A,N) |  |  |
|  |  | 30/05/2020 | ETS(A,Ad,N) | 11/11/2020 | ETS(A,Ad,N) |  |  |
|  |  | 31/05/2020 | ETS(A,A,N) |  |  |  |  |
|  |  | 06/06/2020 | ETS(A,Ad,N) |  |  |  |  |
|  |  | 08/06/2020 | ETS(A,A,N) |  |  |  |  |
|  |  | 10/06/2020 | ETS(A,Ad,N) |  |  |  |  |
|  |  | 12/06/2020 | ETS(A,A,N) |  |  |  |  |
|  |  | 13/06/2020 | ETS(A,Ad,N) |  |  |  |  |
|  |  | 17/06/2020 | ETS(A,A,N) |  |  |  |  |
|  |  | 18/06/2020 | ETS(A,Ad,N) |  |  |  |  |
|  |  | 19/06/2020 | ETS(A,A,N) |  |  |  |  |
|  |  | 20/06/2020 | ETS(A,Ad,N) |  |  |  |  |

****The model alternates the parametrization of ETS(A,A,N) and ETS(A,Ad,N) parametrization until 18-12-2022.**
